# A rare case of human pulmonary dirofilariasis with nodules mimicking malignancy: approach to diagnosis and treatment

**DOI:** 10.1186/s13019-018-0750-5

**Published:** 2018-06-11

**Authors:** Paolo Albino Ferrari, Antonella Grisolia, Stefano Reale, Rosa Liotta, Alessandra Mularoni, Alessandro Bertani

**Affiliations:** 1 Department for the Treatment and Study of Cardiothoracic Diseases and Cardiothoracic Transplantation, IRCCS ISMETT, Via Tricomi 5, Palermo, Italy; 2 Infectious Diseases Unit, IRCCS ISMETT, Via Tricomi 5, Palermo, Italy; 3 Istituto Zooprofilattico Sperimentale della Sicilia Adelmo Mirri, Via Gino Marinuzzi 3, Palermo, Italy; 4 Pathology Service, Department of Diagnostic and Therapeutic Services, IRCCS ISMETT, Via Tricomi 5, Palermo, Italy; 5 Division of Thoracic Surgery, “A. Businco” Oncology Hospital – Azienda Ospedaliera Brotzu, Via Jenner 1, 09100 Cagliari, Italy

**Keywords:** Pulmonary dirofilariasis, Video-assisted thoracic surgery, PCR, Lung nodules

## Abstract

**Background:**

Human pulmonary dirofilariasis is a rare zoonosis caused by the dog worm Dirofilaria spp., a parasite transmitted by mosquitos and resulting in peripheral lung nodules. The filarial nematode enters the subcutaneous tissue, travels to the right ventricle and dies causing a small pulmonary infarction that may embolize through the pulmonary vessels and may appear as a solitary nodule. These nodules are usually incidentally identified in asymptomatic patients undergoing chest imaging studies, and are generally interpreted to be malignant.

**Case presentation:**

We present the case report of a human dirofilariasis in a patient with multiple pulmonary nodules resected using video-assisted thoracic surgery (VATS). According to our literature review, this is the first case with double synchronous lung nodules reported in Italy.

**Conclusions:**

Minimally invasive resection with histologic examination may be the best approach for the diagnosis and treatment of pulmonary dirofilariasis. Polymerase Chain Reaction testing may provide a more accurate etiological diagnosis in case of an inconclusive pathology result.

## Background

Human dirofilariasis is a zoonotic infection mostly caused by the filarial nematodes *Dirofilaria repens* and *Dirofilaria immitis*. It is poorly recognized even in endemic areas such as the Mediterranean and the south of Italy. Dirofilariae are Onchocercidae nematodes that usually target dogs and wild carnivores living in tropical and temperate regions. Humans can be accidentally infected with *Dirofilaria* larvae through mosquito bites [1, 2]. Nodules presenting in parenchymal organs are often misidentified as malignant tumors, requiring biopsy or surgery before being correctly diagnosed [3, 4].

According to our review of the literature, we report the first case of an Italian patient with two pulmonary nodules, mimicking malignant lesions, which were surgically resected and underwent subsequent morphological and molecular identification of *Dirofilaria repens* [5–12]*.*

## Case presentation

**Fig. 1:**
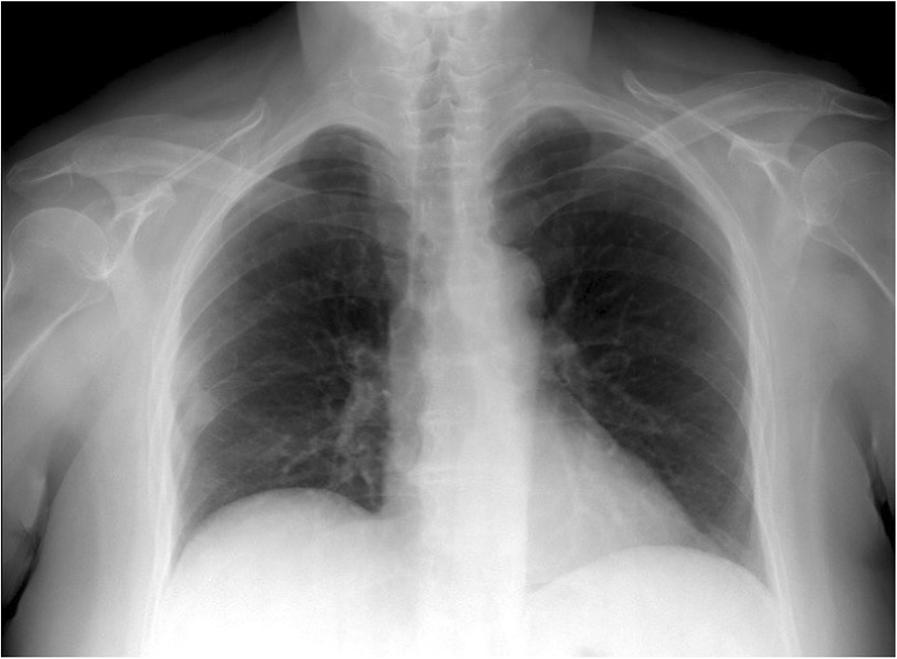
CXR evidence of peripheral right lung nodules

**Fig. 2:**
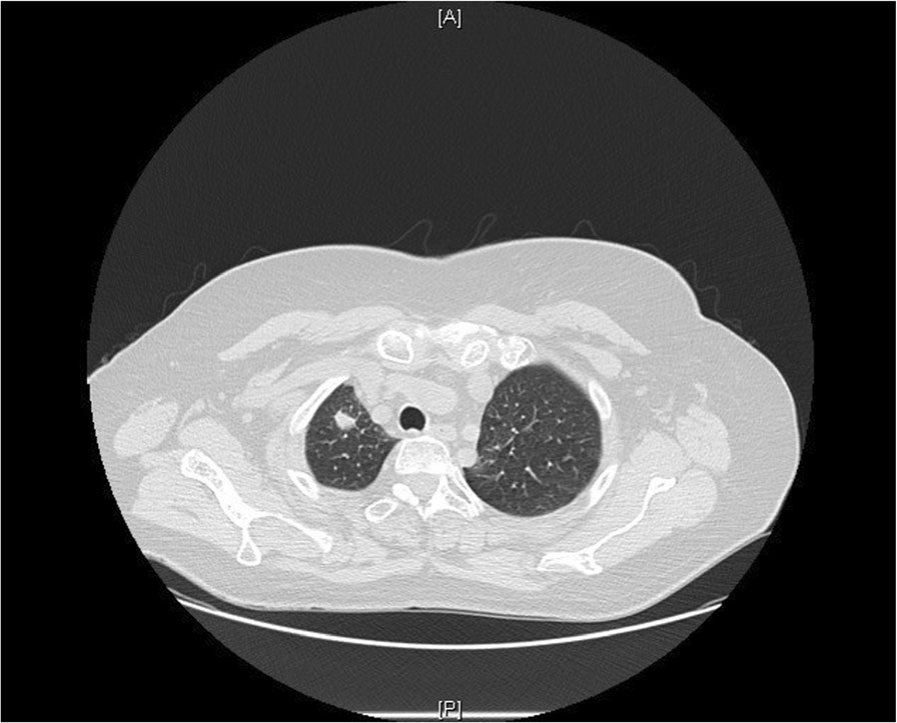
CT scan showing pulmonary right upper lobe nodule

**Fig. 3:**
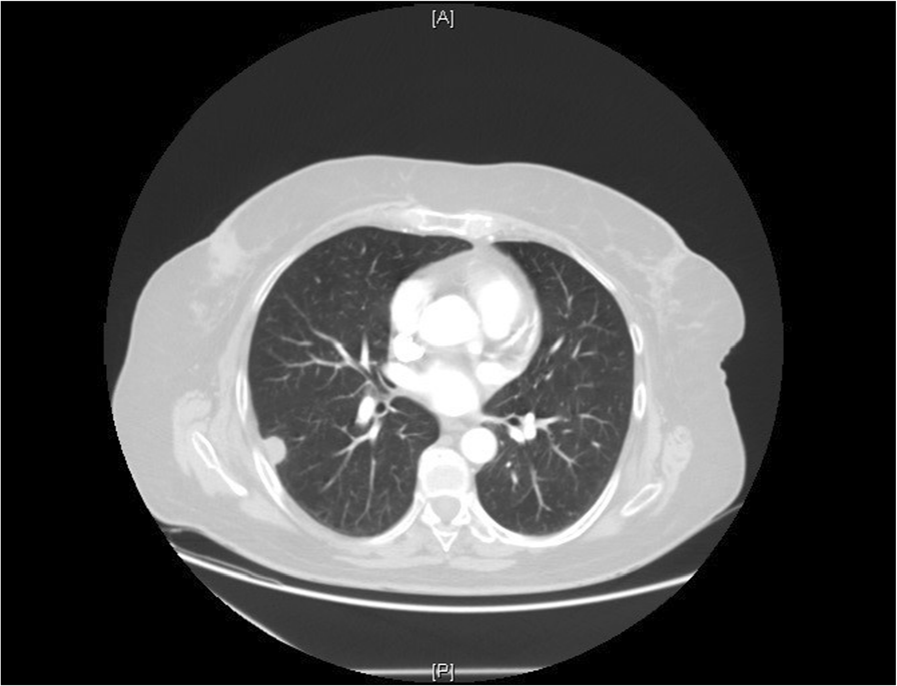
CT scan showing pulmonary right lower lobe nodule

**Fig. 4:**
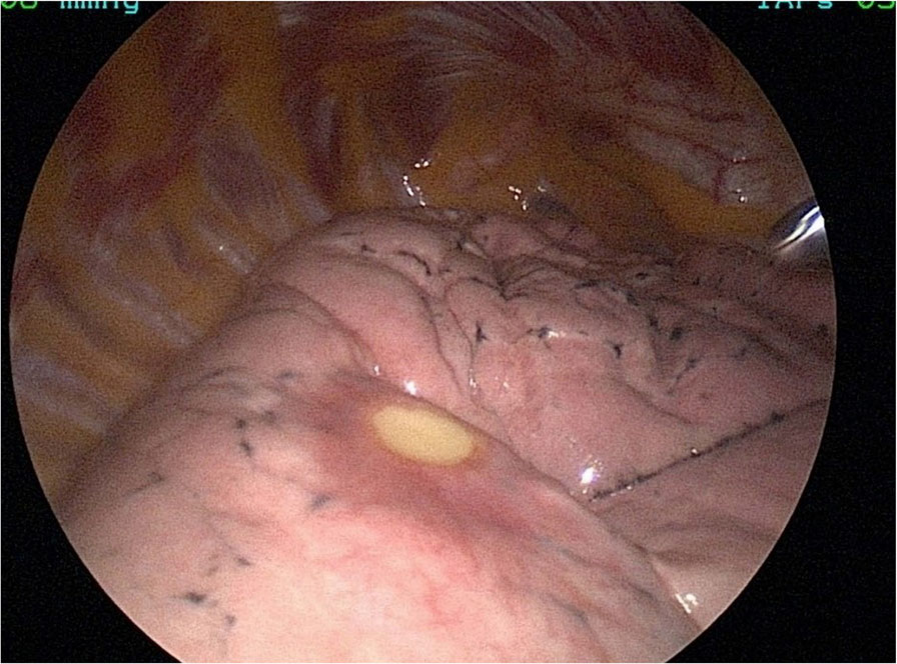
Thoracoscopic evidence of unusual coin pulmonary lesion

**Fig. 5:**
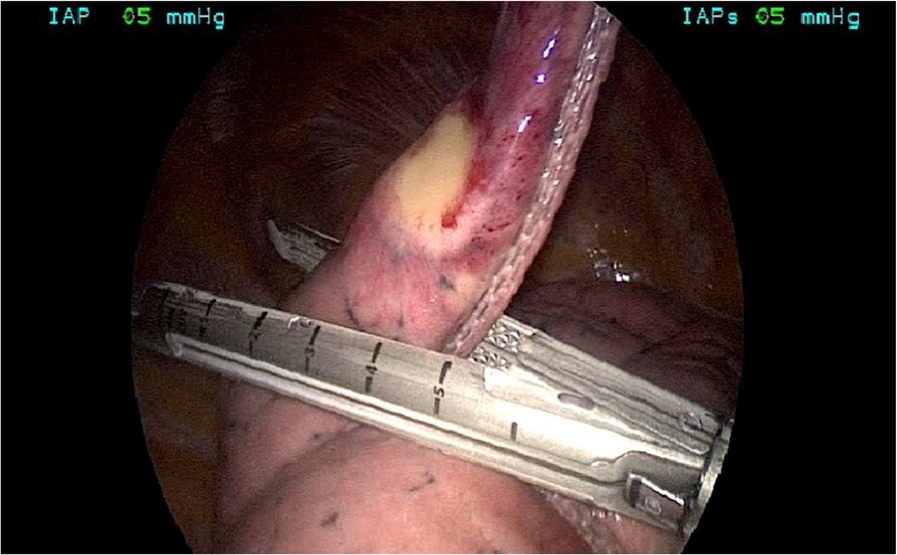
Thoracoscopic pulmonary wedge resection

**Fig. 6:**
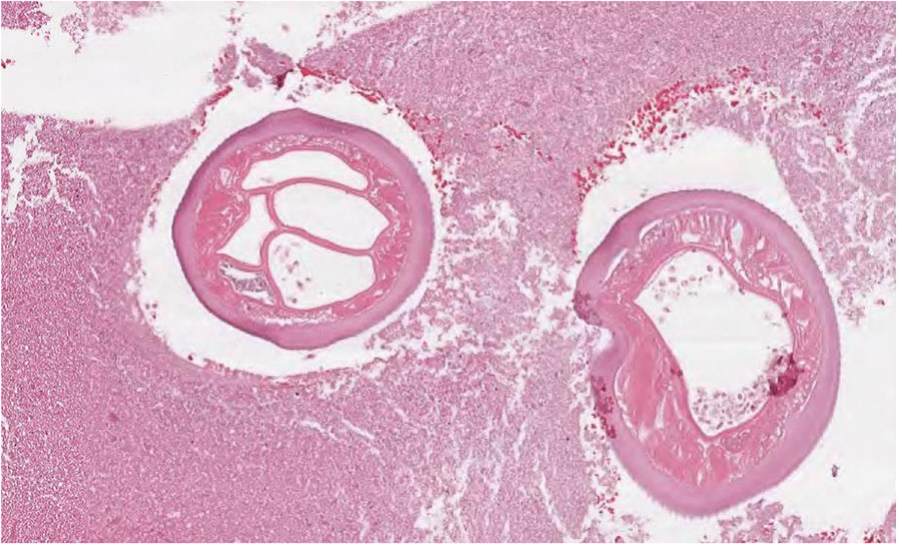
Cross section of *D. repens*

**Fig. 7:**
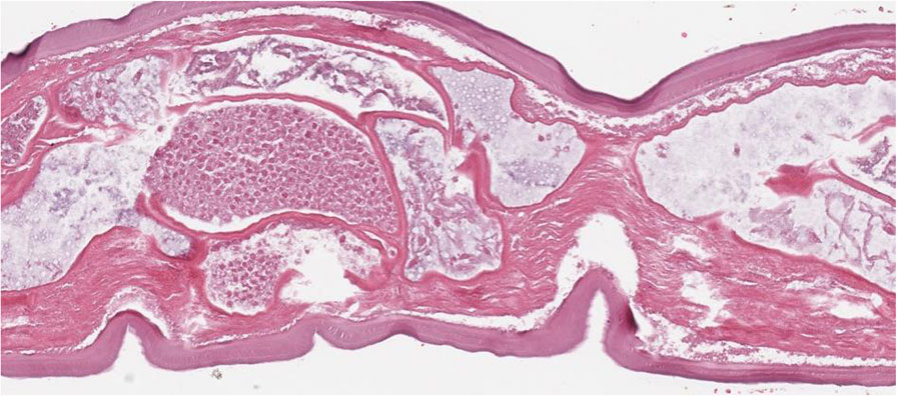
Longitudinal section of *D. repens*

A 63-year-old woman from the south of Italy was admitted to the thoracic surgical service for the evaluation of two coin lesions of the right lung. The lesions were found incidentally on a chest X-ray (Fig. 1) that the patient received for a clinical suspicion of pneumonia. Past medical and surgical history was unremarkable other than for cystocele repair at age 36. Further imaging showed two oval-shaped, non-calcified, well demarcated PET-negative lung lesions measuring approximately 13 × 8 mm in the apical segment of the right upper lobe and in the superior segment of the right lower lobe (Figs. 2, 3). Physical examination of the patient and pulmonary function tests were within normal limits (VC = 108%; FEV1 = 95%). The white blood cell count was 6520/ml without eosinophilia (Eosinophils = 1.2%). A CT-guided needle biopsy of the lesions was performed, showing CD68+ histiocytes, lymphocytes and myofibroblasts suggestive of a benign, granulation-like tissue. Given the inconclusive diagnosis, two wedge pulmonary resections were performed using a minimal invasive approach (Figs. 4, 5). The final pathologic review of the resected lesions suggested a morphological diagnosis of human pulmonary dirofilariasis (Figs. 6, 7). Postoperative serological testing for anti-Filaria antibodies using ELISA was consistent with the above diagnosis. The patient had an uneventful recovery and was discharged home on post-operative day 3. A pharmacological treatment was not considered since complete resection is considered to be curative [5].

**Fig. 8:**
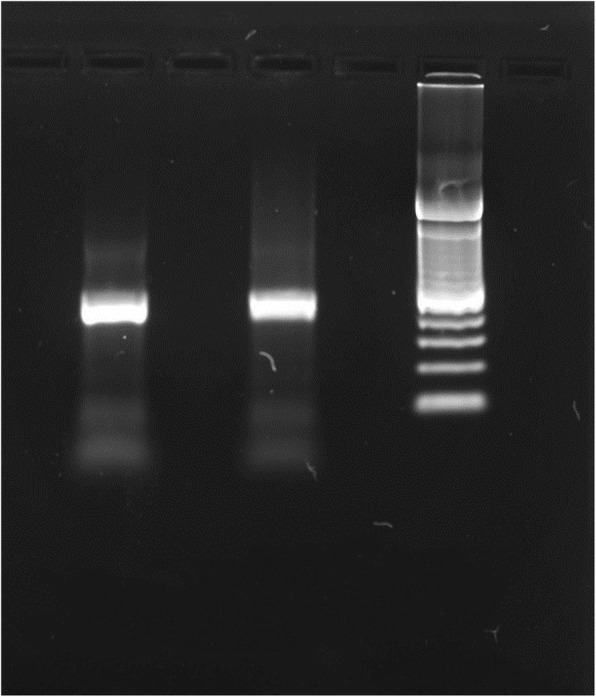
Electrophoresis on 2% agarose gel. Lane 1: 480 bp amplicon obtained from human sample. Lane 2: Negative control. Lane 3: 480 bp amplicon obtained from Dirofilaria repens DNA positive control

A biomolecular testing procedure was performed to further validate the diagnosis, and to discriminate the specific Dirofilaria subspecies. Total DNA extraction was carried out from paraffin embedded tissue taken from the lung nodules, by using a QIAmp DNA mini kit. Reference genomic DNA was extracted from animal blood containing *D.repens* (15,000 mff/ml). A quantity of 20 ng DNA was used for the amplification with four specific forward primers and a common reverse primer as showed in Table 1. The capillary electrophoresis (Fig. 8) was performed on an ABI Prism 3130 DNA sequencer. The collected data was then analyzed, considering 97% identity as the stringent parameter for strain identification. Multiplex-PCR *cox*1 amplicons from the DNA extracted from the embedded tissue resulted in a band consistent with *D. repens (479 bp)*. The BLAST analysis of the *cox*1 sequences revealed a 99 to 100% identity compared to the sequences available in GenBank™.

**Table 1:** DNA amplification structure

PRIMER NAMES	PRIMER SEQUENCES
Arcox1F	5’-ATC TTT GTT TAT GGT GTA TC-3’
Cbcox1F	5’-CGG GTC TTT GTT GTT TTT ATT GC-3’
Dicox1F	5’-ACC GGT GTT TGG GAT TGT TA-3’
Drcox1F	5′-GTA TA TTT TGG GTT TAC ATA CTG TA-3’
Common reverse primer NTR	5′-ATA AGT ACG AGT ATC AAT ATC-3’

## Discussion

Growing concern over *D. repens* in endemic areas of Southern Europe may be justified by the recent, increasing number of published human cases, with an increasing prevalence in Italy (4.5/year between 1986 and 1998; 15.6/year between 1999 and 2009) [5]. Climate changes, insecticide resistance, expanding geographic distribution of both the vectors and the pathogens via modern transportation and globalization are identified as possible causes of the spreading of vector-borne diseases [3]. Human dirofilariasis is currently considered an emergent zoonosis in Italy [7], France [8], Hungary [9] and Russia [10].

Women have a trend to be more commonly infected than men, although without any statistical difference [3, 6]. Twenty-seven cases of pulmonary dirofilariasis have been reported in the medical literature between 1981 and 2010 [3, 6, 11–13]. The differential diagnosis of the visceral localization of disease frequently (but not only) includes malignancy, requiring a biopsy or surgery for a conclusive histologic diagnosis. More recently, the introduction of molecular methods based on polymerase chain reaction and sequencing has improved diagnostic accuracy [14–17].

The use of minimally invasive techniques for the diagnostic and therapeutic excision of lung nodules of unclear origin should be strongly encouraged. The rarity of this zoonosis may also warrant the use of biomolecular assays as a tool to achieve an unequivocal etiological diagnosis and to assist the histo-pathologic and microbiologic diagnostic interpretation.

## Abbreviations

BLAST: Basic Local Alignment Search Tool
CT: Computed Tomography
CXR: Chest X-ray
ELISA: Enzyme-linked Immunosorbent Assay
FEV1%: Forced Expiratory Volume % in the 1st second
PET: Positron Emission Tomography
VC: Vital Capacity
